# Impact of sex in stroke in the young

**DOI:** 10.1371/journal.pone.0274722

**Published:** 2023-03-31

**Authors:** Anina Schwarzwald, Urs Fischer, David Seiffge, Morin Beyeler, Adrian Scutelnic, Johannes Kaesmacher, Pasquale Mordasini, Tomas Dobrocky, Jan Gralla, Mirjam R. Heldner, Roza Umarova, Thomas R. Meinel, Marcel Arnold, Simon Jung, Barbara Goeggel Simonetti

**Affiliations:** 1 Department of Neurology, Inselspital, Bern University Hospital, and University of Bern, Bern, Switzerland; 2 Department of Neurology, Universitätsspital, Basel University Hospital, and University of Basel, Basel, Switzerland; 3 University Institute of Diagnostic and Interventional Neuroradiology, Inselspital, Bern University Hospital, and University of Bern, Bern, Switzerland; 4 Institute of Diagnostic and Interventional Neuroradiology, Cantonal Hospital, St. Gallen, Guangdong Province, China; 5 Division of Neuropediatrics, Institute of Pediatrics of Southern Switzerland, Bellinzona, and Università della Svizzera Italiana, Lugano, Switzerland; UMMC: The University of Mississippi Medical Center, UNITED STATES

## Abstract

**Background and purpose:**

Limited data is available on sex differences in young stroke patients describing discrepant findings. This study aims to investigate the sex differences in young stroke patients.

**Methods:**

Prospective cohort study comparing risk factors, etiology, stroke localization, severity on admission, management and outcome in patients aged 16–55 years with acute ischemic stroke consecutively included in the Bernese stroke database between 01/2015 to 12/2018 with subgroup analyses for very young (16-35y) and young patients (36-55y).

**Results:**

689 patients (39% female) were included. Stroke in women dominated in the very young (53.8%, p<0.001) and in men in the young (63.9%, p<0.001). As risk factors only sleep-disordered breathing was more predominant in men in the very young, whereas arterial hypertension, diabetes and atrial fibrillation did not differ in women and men older than 35y. The higher frequency of stroke in women in the very young may be explained by the sex specific risk factors such as pregnancy, puerperium, the use of oral contraceptives, and hormonal replacement therapy. Stroke severity at presentation, etiology, stroke localization, management, and outcome did not differ between women and men.

**Conclusions:**

The main finding of this study is that sex specific risk factors in women may contribute to a large extent to the higher incidence of stroke in the very young in women. Important modifiable stroke risk factors, such as arterial hypertension, diabetes mellitus and atrial fibrillation did not differ in women and men, either in the young as well as in the very young. These findings have major implications for primary preventive strategies of stroke in young people.

## Introduction

According to the World Health Organization (WHO), stroke is the second leading cause of death worldwide. It is also one of the most common causes of disability [1]. Young adults up to the age of 50 account for about 10% of all stroke patients [2]. The incidence of ischemic stroke among the young is increasing globally [3]^.^ Despite an overall good prognosis of stroke in young people, there is an important socioeconomic impact [3, 4].

Sex differences in stroke are well known, in that women are older at their first stroke and suffer poorer outcome [5]. However, only a few studies have addressed the sex differences of stroke in young. It is unknown if risk factors unique to women, such as pregnancy and puerperium, and the use of oral contraceptives account for the higher stroke incidence among women in the very young stroke patients (<35 years) reported by some studies [3, 6–8]^.^ Only a few studies addressed the difference in the risk factors among both the sexes. Arterial hypertension, diabetes mellitus and smoking seem to be more prevalent in men while low physical activity and high body mass index (BMI) are more prevalent in women [7, 9]. The evidence on differences in etiology, localization and outcome between the sexes in young stroke patients is scarce. A better understanding of sex differences provides a further step towards individualized medicine. This study aims to determine the differences in stroke manifestation in young people of either sexes with regard to the severity of the stroke presentation, risk factors, etiology, infarct localization, acute therapy and outcome stratified according to age groups.

## Methods

This is a prospective, single-center cohort study, with consecutive patients, who gave their general consent for using their personal data for research purposes, with acute ischemic stroke (AIS), transient ischemic attack (TIA), retinal infarction or transient monocular blindness aged between 16 and 55 years, who presented at the Stroke Center of the University Hospital Inselspital Bern between January 2015 and December 2018. The cut-off of 55 years has been chosen based on current expert recommendations [10]. Only patients who gave consent to use their health related data for research purposes have been included. The following variables were collected prospectively: Demographic data, data on past medical history, National Institutes of Health Stroke Scale (NIHSS) [11] on admission, information about acute treatment (antiplatelet drugs, anticoagulants, intravenous thrombolysis (IVT) with recombinant tissue plasminogen activator (rtPA) and intra-arterial treatment (IAT) (including pharmacological, such as fibrinolysis by rtPA or urokinase, and mechanical treatment), risk factors, etiology (according to the TOAST (Trial of Org 10172 Acute Stroke Treatment) classification [12]), infarct localization (divided into anterior and posterior vascular territory), outcome after three months according to a modified Rankin Scale score (mRS) [13], and recurrent events.

The study was approved by the institutional review board, the ethics committee of Bern, and conducted in accordance with institutional guidelines.

Ethical approval and consent to participate in form of general consent, as required by the local regulations, was given in written form for all participants. The consent of minors was obtained from their parents or guardians.

### Definition and data acquisition of specific variables

Persistent foramen ovale (PFO) was diagnosed by transesophageal echocardiography with adequate Valsalva maneuver. Screening for sleep-disordered breathing was performed by ApneaLink™ (by ResMed), and in case of relevant findings validated by polysomnography.

The etiology of stroke was classified according to the TOAST classification [12]. Each patient received an extensive etiologic work-up including cerebral imaging (computed tomography angiography (CTA) and/or magnetic resonance angiography (MRA)), transesophageal echocardiography, at least 3 weeks of long-term ECG, and hematological work-up. If a stroke was only associated with a PFO as a possible cause the stroke’s etiology was classified as "undetermined". The anterior circulation stroke was defined as stroke located in the Middle Cerebral and Anterior Cerebral Artery territories and the posterior circulation stroke comprised stroke in the Posterior Cerebral Artery, Brainstem and Cerebellum territories.

### Statistics

The statistical analysis was performed with IBM SPSS® version 25. Fisher’s exact test or Pearson χ2 test were used for categorical variables and Mann-Whitney U test for continuous variables. We performed subgroup analysis for the 16–35 and 36–55 year age groups. A two-tailed probability value < 0.05 was considered statistically significant.

## Results

### Demographics

Between January 2015 and December 2018, 689 consecutive patients were included in this analysis: 554 AIS (80.4%), 126 TIA (18.3%), six transient monocular blindness (0.9%) and three with retinal infarction (0.4%). Overall, there were 419 men (60.8% of 689) and 270 women (39.2%). Among the very young patients aged 16–35, 64 were women (53.8% of 119) and 55 men (46.2%).

### Risk factors

In our study cohort, modifiable risk factors were more prevalent in men, such as dyslipidemia (119 women (45.4%) vs. 238 men (57.9%), p-value = 0.002), smoking (87 women (33.1%) vs. 184 men (45.0%), p-value = 0.002), coronary heart disease (5 women (1.9%) vs. 27 men (6.5%), p-value = 0.005), overweight (BMI ≥ 25 kg/m^2^; 57 women (21.4%) vs. 167 men (40.3%), p-value < 0.001), and sleep-disordered breathing (18 women (10.2%) vs. 71 men (26.5%), p-value < 0.001).

Family history of stroke and/or coronary heart disease, as well as diabetes mellitus did not differ (Table 1A). Although no sex difference was found for persistent foramen ovale, women had more often a Risk of Paradoxical Embolism (RoPE)-score [14] > 7 than men (23 of 61 documented RoPE-Scores > 7 in women (37.7%) vs. 18 of 107 in men (16.8%), p = 0.005).

**Table 1:** A. Risk factors stratified according to sex. **B.** Risk factors stratified according to sex in very young AIS patients (16-35y). **C.** Risk factors stratified according to sex in young AIS patients (36-55y).

**A**				
**Variable/ Characteristic**	**Total n with con-dition/valid n (%)**	**Female n = 270 (%)**	**Male n = 419 (%)**	**p-value**
***Modifiable risk factors***	** **	** **	** **	** **
Dyslipidemia	357/673 (53.0)	119/262 (45.4)	238/411(57.9)	0.002
Smoking	271/672 (40.3)	87/263 (33.1)	184/409 (45.0)	0.002
Arterial hypertension	236/689 (34.3)	80/270 (29.6)	156/419 (37.2)	0.048
Underweight *	17/689 (1.2)	9/270 (3.3)	8/419 (1.9)	0.3
Overweight Ɨ	224/680 (32.9)	57/266 (21.4)	167/414 (40.3)	< 0.001
Obesity ǂ	140/646 (21.7)	61/257 (23.7)	79/389 (20.3)	0.4
Sleep-disordered breathing	89/444 (20.0)	18/176 (10.2)	71/268 (26.5)	< 0.001
Harmful use of alcohol	52/514 (10.1)	12/195 (6.2)	40/319 (12.5)	0.02
Other substance abuse	35/514 (6.8)	4/194 (2.1)	31/320 (9.7)	0.001
Diabetes mellitus	58/682 (8.5)	23/265 (8.7)	35/417 (8.4)	0.9
History of stroke	59/680 (8.7)	16/264 (6.1)	43/416 (10.3)	0.07
History of TIA	34/546 (6.2)	14/223 (6.3)	20/323 (6.2)	1.00
Atrial fibrillation	21/688 (3.1)	7/270 (2.6)	14/418 (3.3)	0.7
CHD	32/688 (4.7)	5/270 (1.9)	27/418 (6.5)	0.005
PFO	197/488 (40.4)	74/192 (38.5)	123/296 (41.6)	0.6
Pregnancy/Puerperium		13/270 (4.8)	** **	** **
Contraceptives or HRT		35/270 (5.1)		
***Non modifiable risk factors***				
Family history of stroke	118/538 (21.9)	48//213 (22.5)	70/325 (21.5)	0.8
Family history of CHD	116/521 (22.3)	49/206 (23.8)	67/315 (21.3)	0.5
**B**				
**Variable/ Characteristic**	**Total n with con-dition/valid n (%)**	**Female (n = 64)**	**Male (n = 55)**	**two-tailed p-value**
***Modifiable risk factors***	** **	** **	** **	** **
Arterial hypertension	9/119 (7.6)	3/64 (4.7)	6/55 (10.9)	0.3
Diabetes mellitus	1/116 (0.9)	0/61 (0.0)	1/55 (1.8)	0.5
Dyslipidemia	35/116 (30.2)	16/62 (25.8)	19/54 (35.2)	0.3
Smoking	39/17 (33.3)	17/63 (27.0)	22/54 (40.7)	0.1
Atrial fibrillation	0/119 (0.0)	0/64 (0.0)	0/55 (0.0)	1.0
CHD	0/119 (0.0)	0/64 (0.0)	0/55 (0.0)	1.0
History of stroke	10/116 (8.6)	5/61 (8.2)	5/55 (9.1)	1.0
History of TIA	6/96 (6.3)	3/52 (3.1)	3/44 (3.1)	1.0
Underweight*	5/113 (4.4)	3/61 (4.9)	2/52 (3.8)	1.0
Overweight Ɨ	29/113 (25.7)	13/61 (21.3)	16/52 (30.8)	0.3
Obesity ǂ	17/113 (15.0)	6/61 (9.8)	11/52 (21.2)	0.1
Sleep-disordered breathing	7/82 (8.5)	0/40 (0.0)	7/42 (16.7)	0.01
Alcohol overuse	1/89 (1.1)	0/48 (0.0)	1/41 (2.4)	0.5
Substance abuse	3/89 (3.4)	0/48 (0.0)	3/41 (7.3)	0.09
PFO	36/80 (45.0)	17/39 (43.6)	19/41 (46.3)	0.9
Pregnancy/Puerperium		12/61 (19.6)		
Contraceptives or HRT		15/61 (24.6)		
***Non modifiable risk factors***				
Family history of stroke	22/102 (21.6)	14/57 (24.6)	8/45 (17.8)	0.5
Family history of CHD	15/100 (15.0)	7/55 (12.7)	8/45 (17.8)	0.6
**C**				
**Variable/ Characteristic**	**Total n with con-dition/valid n (%)**	**Female (n = 206)**	**Male (n = 364)**	**two-tailed p-value**
***Modifiable risk factors***				
Arterial hypertension	227/570 (39.8)	77/206 (37.4)	150/364 (41.2)	0.4
Diabetes mellitus	57/566 (10.1)	23/204 (11.3)	34/362 (9.4)	0.5
Dyslipidemia	322/557 (57.8)	103/200 (51.5)	219/357 (61.3)	0.03
Smoking	232/555 (41.8)	70/200 (35.0)	162/355 (45.6)	0.02
Atrial fibrillation	21/569 (3.7)	7/206 (3.4)	14/363 (3.9)	1.0
CHD	32/569 (5.6)	5/206 (2.4)	27/363 (7.4)	0.01
History of stroke	49/564 (8.7)	11/203 (5.4)	38/361 (10.5)	0.04
History of TIA	30/460 (6.5)	11/169 (6.5)	19/291 (6.5)	1.0
Underweight *	12/527 (2.3)	6/193 (3.1)	6/334 (1.8)	0.4
Overweight Ɨ	197/527 (37.4)	44/193 (22.8)	153/334 (45.8)	<0.001
Obesity ǂ	123/527 (23.3)	55/193 (28.5)	68/334 (20.4)	0.04
Sleep-disordered breathing	82/421 (19.5)	18/152 (11.8)	64/269 (23.8)	0.003
Alcohol overuse	51/425 (12.0)	12/147 (8.2)	39/278 (14.0)	0.09
Substance abuse	32/425 ((7.5)	4/146 (2.7)	28/279 (10.0)	0.006
PFO	161/408 (39.5)	57/153 (37.3)	104/255 (40.8)	0.5
Pregnancy/Puerperium		1/206 (0.5)		
Contraceptives or HRT		20/206 (9.7)		
***Non modifiable risk factors***				
Family history of stroke	96/436 (22.0)	34/156 (21.8)	62/280 (22.1)	1.0
Family history of CHD	101/421 (24.0)	42/151 (27.8)	59/270 (21.9)	0.2

TIA indicates transient ischemic attack; PFO, persistent foramen ovale; HRT, hormone replacement therapy; CHD, coronary heart disease

Body Mass Index (BMI)

* BMI < 18.5 kg/m2

Ɨ BMI < 30 and ≥ 25 kg/m2

ǂ BMI ≥ 30 kg/m2

The sex specific risk factors pregnancy and puerperium were found in 13/270 women (4.8%), and 35 (13%) were users of oral contraceptives or hormone replacement therapy on admission.

**Fig 1:**
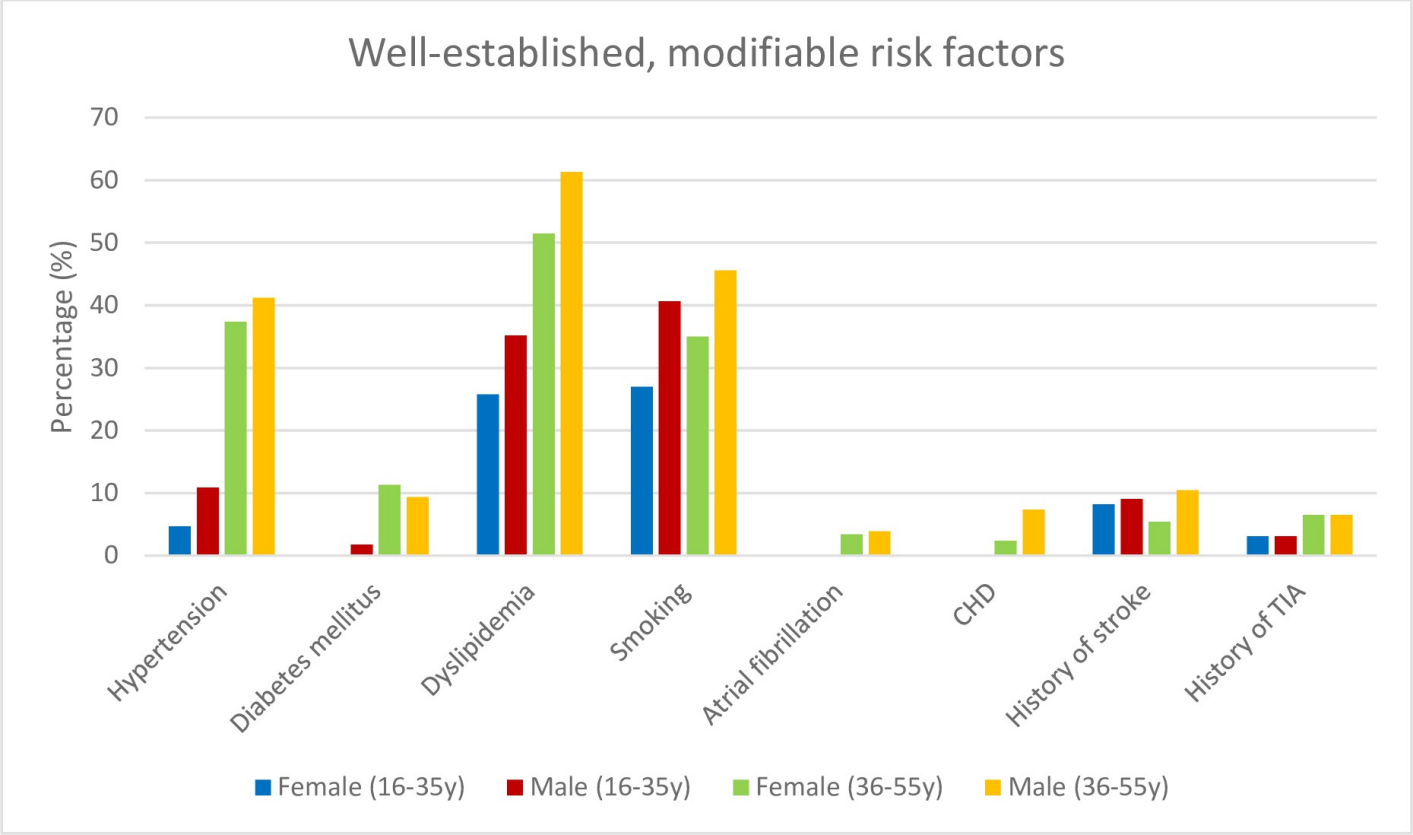
Y indicates years; CHD, coronary heart disease; TIA, transient ischemic attack.

The subgroup analysis of very young patients, i.e 16–35 years old, did not demonstrate any significant difference in the prevalence of modifiable risk factors between both the sexes, except for sleep-disordered breathing (0 women vs. 7 men (16.7%), p-value = 0.01) and the sex specific risk factors pregnancy and puerperium in women. In 36-55-year-old patients, the prevalence of arterial hypertension, diabetes mellitus, atrial fibrillation, and PFO did not differ between women and men (Table 1C). In this age group, the remainder risk factors were more frequent in men (Table 1C and Fig 1).

### Stroke etiology

**Fig 2:**
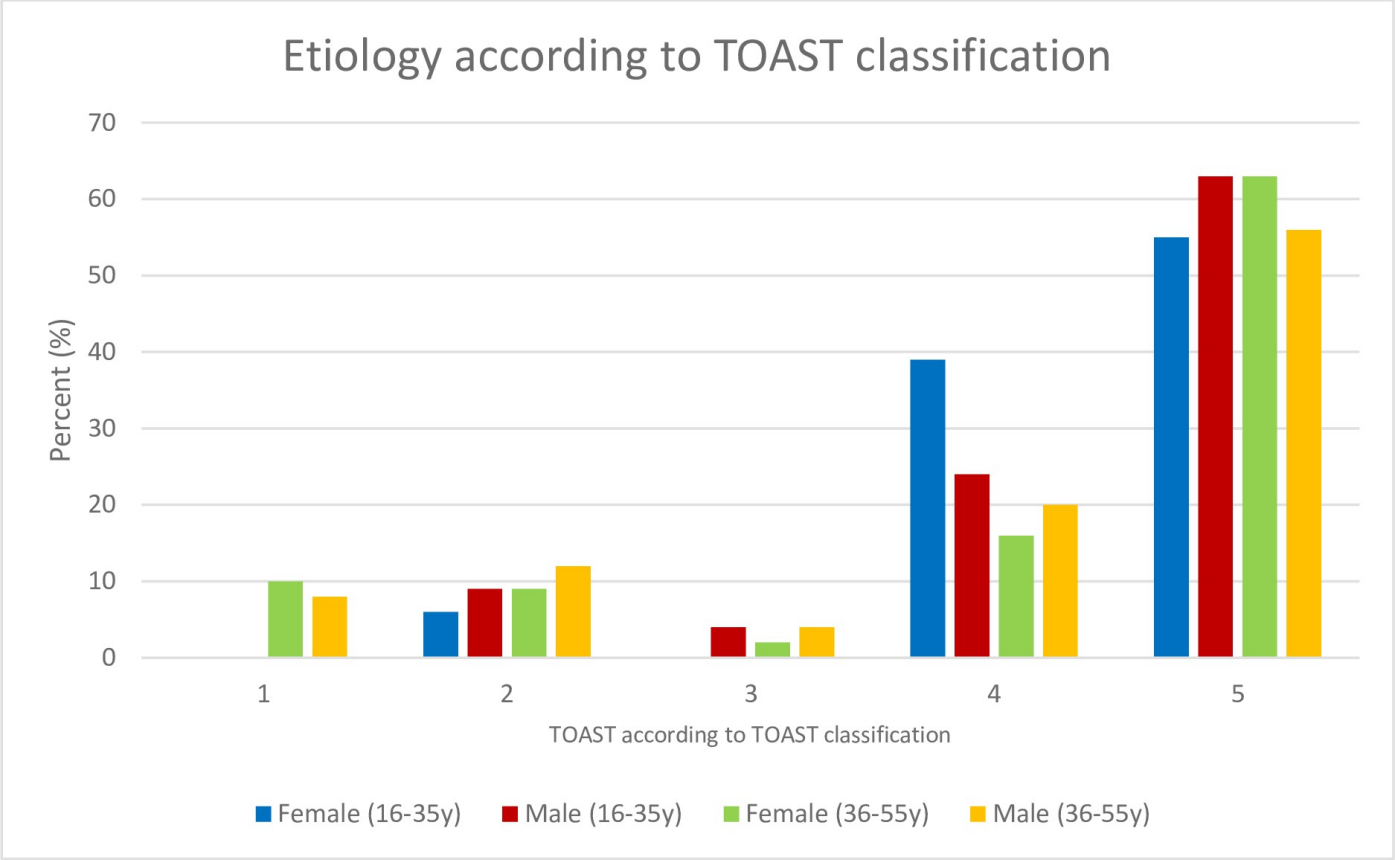
Y indicates years. 1: Large artery atherosclerosis/macroangiopathy. 2: Cardiac embolism. 3: Small vessel disease/microangiopathies. 4: Other determined etiology. 5. Undetermined etiology, including undetermined despite complete evaluation, undetermined without complete evaluation and multiple possible etiologies.

Stroke etiology did not differ between the sexes, neither in the whole cohort nor within the two age groups. Of note, the cause of stroke in young adults remained most often undetermined (403 (58.7%) of 687, 164 of 269 women (60.9%) vs. 239 of 418 men (57.2%)). Stroke due to cardioembolism was found in 70 patients (of 687, 10.2%, 23 of 269 women (8.6%) vs. 47 of 418 men (11.2%)). Other determined etiologies were found in 143 (20.8% of 687, 57 women (21.2%) vs. 86 men (20.6%)). (Fig 2).

### Infarct localization

Men and women did not differ in terms of stroke localization, neither in the whole study population, nor in the two age groups (175 of 270 women (65%) and 282 of 419 men (67%) with anterior circulation stroke: 95 women (35%) and 137 men (33%) with posterior circulation stroke (p = 0.5)). In addition, stroke localization in the age subgroups did not differ (anterior circulation in 58% of women and 62% of men in the 16-35year age group (p = 0.7); 67% of women and 68% of men in the 36-45year age group (p = 0.8)).

### Stroke severity on admission and acute treatment

Stroke severity on admission was measured by the NIHSS and ranged from 0–23 in women and from 0–42 in men. Overall, there was no difference between the sexes (mean NIHSS women: 4.61, men: 4.65, p = 0.56), nor the choice of acute treatment (Table 2).

**Table 2:** Treatment procedures according to sex.

	Valid n/total (t = 689) (%)	Female (t = 270) (%)	Male (t = 419) (%)	Two-tailed p-value
Antiplatelet drugs	387 (56.2)	154 (57.0)	233 (55.6)	0.8
Anticoagulants	48 (7.0)	17 (6.3)	31/419 (7.4)	0.6
IVT with rTPA	126 (18.3)	40 (14.8)	86 (20.5)	0.07
IAT	138 (20.0)	54 (20.0)	84 (20.0)	1.0

IVT indicates intravenous thrombolysis; rtPA, recombinant tissue plasminogen activator; IAT, intra-arterial treatment

### Clinical outcome after three months

A majority of both men (87%) and women (87%) had a good outcome defined as a mRS ≤ 2 (p = 0.8). Case fatality did not differ (4.0% in women and 4.6% in men, p = 0.84), nor did recurrent ischemic events (4.4% in women vs. 2.9% in men, p = 0.39).

## Discussion

The main finding of our study is that men have a higher incidence of sleep-disordered breathing in the 16–35 years old stroke patients, but the higher incidence of stroke in women in this age group may largely be explained by sex specific risk factors, including pregnancy, puerperium and the use of oral contraceptives. In patients older than 35, risk factors such as arterial hypertension, diabetes mellitus and atrial fibrillation did not differ between women and men. We did not find sex differences regarding stroke etiology, severity at presentation, localization, and outcome in stroke in the young.

In accordance with previous studies, we found a female predominance in the age group 16–35 in contrast to a male predominance in the age group 36–55 [7, 8, 15–17].

### Risk factors

A male predominance in risk factors such as dyslipidemia, smoking, arterial hypertension, coronary heart disease, substance and alcohol abuse was demonstrated in the presented cohort. This is in line with previous studies [2, 3, 7, 9, 17, 18]. While overweight was more frequent in men, women had a higher obesity rate. While some studies report higher obesity rates in female young stroke patients [3, 9, 19], the Helsinki Young Stroke Registry found no sex difference regarding obesity [7].

The subgroup analysis in patients aged 36–55 years revealed no differences regarding the most important risk factors such as arterial hypertension, diabetes mellitus and atrial fibrillation. In the 16–35 year age group the sexes only differed in sleep-disordered breathing, and the female sex specific risk factors. Sleep-disordered breathing was more prevalent in men. A male predominance of sleep-disordered breathing was also found in the sifap1 study [19] and the Helsinki Young Stroke Registry, but both did not further analyze different age groups in the young [7].

The sex specific risk factors, such as pregnancy and puerperium, were almost exclusively present in the age group 16–35. This may to some extent explain the higher incidence of stroke in women in childbearing age [15, 20–22].

### Stroke etiology

Unlike prior reports, we did not find sex specific differences in stroke etiology classified by TOAST criteria. Previous studies described a higher rate of large artery atherosclerosis, [7, 17, 18] and small vessel disease [7, 17, 23] in young men compared to young women, while stroke in women was more often classified as “other determined” or undetermined cause [17, 18, 23, 24]. In accordance with previous studies we found a higher rate of undetermined stroke etiologies in young stroke patients despite an extensive etiological work-up.

The finding of a higher prevalence of stroke risk factors in young men likely explains that women tend to have a higher RoPE-score (> 7) than men. Future studies should therefore address whether stroke in the young is more often due to paradox embolism in women than in men. Given the higher RoPE-scores in women, it should be addressed, whether women might benefit more from a mechanical closure of the PFO.

### Stroke severity, infarct localization, treatment, and outcome

Neither stroke severity expressed by the NIHSS, nor the affected vascular territory differed between sexes. In the absence of a significant difference in etiology, stroke severity and localization, it is not surprising that no sex-related differences in the applied treatment modalities and the three-month clinical outcome expressed with the mRS was demonstrated. Only one previous study analyzed sex differences in the localization of stroke in young patients, reporting more supratentorial strokes in women [25].

Female sex was a predictor of worse outcome (mRS>2) at discharge in patients aged 31–50 years after a first ever AIS in a Spanish study [26], while age and male sex were associated with other arterial events, but not with stroke in the FUTURE study [23].

### Limitations

This study is not population-based. Only patients from the area covered by our Stroke Center were included. However, in our region most young stroke patients are admitted to our stroke center, thus decreasing the risk for a selection bias due to the tertiary care setting. An ethnic bias must also be taken into account, since >95% of the population in the surrounding of the Bernese Stroke Center is Caucasian, and hardly any Africans/Asians.

We did not consider socioeconomic factors in our study, thus limiting the analysis of sex aspects, nor did we include patient centered outcome measures such as quality of life.

Our aim was to identify potential differences using univariate comparisons without adjustment for multiple testing. We did not perform multiple testing due to the limited patients of the subgroups, especially of the youngest, and the non-population-based study design. Hence, our findings should be regarded as hypothesis generating and need to be replicated in a population-based larger study.

In order to determine further characteristics of young stroke patients, a control group of young adults without stroke would be necessary. In this respect, a preliminary comparison of our cohort group with peers in the general Swiss population was made. The characteristics of the latter are regularly assessed by national surveys. The results are attached in the supplemental material.

## Supporting information

S1 File (XLSX)

S2 File (XLSX)

S1 Data (DOCX)

## Abbreviations and acronyms

AIS: acute ischemic stroke
IAT: intra-arterial treatment
IVT: intravenous thrombolysis
mRS: modified Rankin Scale
NIHSS: National Institutes of Health Stroke Scale
PFO: persistent foramen ovale
RoPE: risk of paradoxical embolism
rtPA: recombinant tissue plasminogen activator
TIA: transient ischemic attack
TOAST: Trial of Org 10172 in Acute Stroke Treatment
