# Supplementary material for: Impact of sex in stroke in the young
Source: PLoS One. 2023 Mar 31;18(3):e0274722. doi: 10.1371/journal.pone.0274722 (PMC10065293; doi:10.1371/journal.pone.0274722)
Supplement: S1 Data — (DOCX) [file pone.0274722.s003.docx]

**Supplemental material**

We compared the risk factor prevalence in our study population with the age matched general Swiss population based on data of the National Health Survey [1].

**Tab. 3:** Risk factors according to sex in study and general Swiss population

|  | | **Study population (age 36-55y)** | | | | **General Swiss population (age 35-54y) *** | |
| --- | --- | --- | --- | --- | --- | --- | --- |
| **Traditional risk factors** | | **Female n=270 (%)** | **Male n=419 (%)** | | | **Female (%)** | **Male (%)** |
| Arterial hypertension | | 37.4 | 41.2 | | | 12.7 - 20.0 | 18.7 - 27.1 |
| Dyslipidemia | | 57.8 | 61.3 | | | 8.7 - 13.7 | 11.6 - 22.0 |
| Smoking | | 35.0 | 45.6 | | | 24.6 - 26.6 | 30.6 - 36.7 |
| Diabetes | | 11.3 | 9.4 | | | 3.0 - 3.1 | 1.0 - 3.6 |
| Overweight | | 22.8 | 45.8 | | | 19.3 - 24.0 | 39.9 - 42.9 |
| Obesity | 28.5 | | | 20.4 | 9.1 - 12.1 | | 11.0 - 14.4 |

* According to the Swiss Federal Statistical Office [1]

Y indicates years

As illustrated in Table 3, the overall prevalence of risk factors in our cohort was higher than in the general population [1], which is also consistent with previous reports [2, 3].

When comparing the frequencies of traditional risk factors in the general Swiss population based on the Health Survey of 2017 published by the Swiss Federal Statistical Office with our study population, it should be noted that the data assessment is methodologically different.

**References**

[1] Bundesamt für Statistik, ‘Gesundheitsverhalten’.

[2] A. Aigner, U. Grittner, A. Rolfs, B. Norrving, B. Siegerink, and M. A. Busch, ‘Contribution of Established Stroke Risk Factors to the Burden of Stroke in Young Adults’, *Stroke*, vol. 48, no. 7, pp. 1744–1751, 2017.

[3] B. Goeggel Simonetti *et al.*, ‘Risk factors, aetiology and outcome of ischaemic stroke in young adults: the Swiss Young Stroke Study (SYSS)’, *J. Neurol.*, vol. 262, no. 9, pp. 2025–2032, 2015.
